# Mann iteration for monotone nonexpansive mappings in ordered CAT(0) space with an application to integral equations

**DOI:** 10.1186/s13660-018-1925-2

**Published:** 2018-12-11

**Authors:** Izhar Uddin, Chanchal Garodia, Juan Jose Nieto

**Affiliations:** 1 Department of Mathematics, Faculty of Natural Sciences, Jamia Millia Islamia, New Delhi, India; 2 Departamento de Estadística, Análisis Matemático y Optimización, Facultad de Matemáticas, Universidad de Santiago de Compostela, Santiago de Compostela, Spain

**Keywords:** 47H09, 47H10, $\operatorname{CAT}(0)$ space, Fixed point, Δ-convergence, Monotone nonexpansive mapping

## Abstract

In this paper, we establish some convergence results for a monotone nonexpansive mapping in a $\operatorname{CAT}(0)$ space. We prove the Δ- and strong convergence of the Mann iteration scheme. Further, we provide a numerical example to illustrate the convergence of our iteration scheme, and also, as an application, we discuss the solution of integral equation. Our results extend some of the relevant results.

## Introduction

The Banach contraction principle [1] is one of the most fundamental results in fixed point theory and has been utilized widely for proving the existence of solutions of different nonlinear functional equations. In the last few years, many efforts have been made to obtain fixed points in partially ordered sets. In 2004, Ran and Reurings [2] generalized the Banach contraction principle to ordered metric spaces. Later on, in 2005, Nieto and Rodriguez [3] used the same approach to further extend some more results of fixed point theory in partially ordered metric spaces and utilized them to study the existence of solutions of differential equations.

Note that the Banach contraction principle is no longer true for nonexpansive mappings, that is, a nonexpansive mapping need not admit a fixed point on a complete metric space. Also, Picard iteration need not converge for a nonexpansive map in a complete metric space. This led to the beginning of a new era of fixed point theory for nonexpansive mappings by using geometric properties. In 1965, Browder [4], Göhde [5], and Kirk [6] gave three basic existence results for nonexpansive mappings. With a view to locating fixed points of nonexpansive mappings, Mann [7] and Ishikawa [8] introduced two basic iteration schemes.

Now, fixed point theory of monotone nonexpansive mappings is gaining much attention among the researchers. Recently, Bachar and Khamsi [9], Abdullatif et al. [10], and Song et al. [11] proved some existence and convergence results for monotone nonexpansive mappings. Dehaish and Khamsi [12] proved the weak convergence of the Mann iteration for a monotone nonexpansive mapping. In 2016, Song et al. [11] considered the weak convergence of the Mann iteration scheme for a monotone nonexpansive mapping *T* under some mild different conditions in a Banach space.

The aim of this paper is to study the convergence behavior of the well-known Mann iteration [7] in a $\operatorname{CAT}(0)$ space for a monotone nonexpansive mapping. Further, we provide a numerical example and application related to solution of an integral equation. Our results generalize and improve several existing results in the literature.

## Preliminaries

To make our paper self-contained, we recall some basic definitions and relevant results.

A metric space *X* is a $\operatorname{CAT}(0)$ space if it is geodesically connected and if every geodesic triangle in *X* is at least as thin as its comparison triangle in the Euclidean plane. For further information about these spaces and the fundamental role they play in various branches of mathematics, we refer to Bridson and Haefliger [13] and Burago et al. [14]. Every convex subset of Euclidean space $\mathbb{R}^{n}$ endowed with the induced metric is a $\operatorname{CAT}(0)$ space. Further, the class of Hilbert spaces are examples of $\operatorname{CAT}(0)$ spaces.

The fixed point theory in $\operatorname{CAT}(0)$ spaces is gaining attention of researchers, and many results have been obtained for single- and multivalued mappings in a $\operatorname{CAT}(0)$ space. For different aspects of fixed point theory in $\operatorname{CAT}(0)$ spaces, we refer to [15–24]. The following few results are necessary for our subsequent discussion.

### Lemma 2.1

([21])

*Let* $(X, d)$ *be a* $\operatorname{CAT}(0)$ *space*. *For* $e, f \in X$ *and* $z \in [0, 1]$, *there exists a unique* $h\in [e, f]$ *such that*

$$\begin{aligned} d(e, h)=zd(e, f) \quad \textit{and}\quad d(f, h)=(1-z)d(e, f). \end{aligned}$$

We use the notation $(1-z)e\oplus z f$ for the unique point *h* of the lemma.

### Lemma 2.2

([21])

*Let* $(X, d)$ *be a* $\operatorname{CAT}(0)$ *space*. *For* $e,f,h \in X$ *and* $z\in [0, 1]$, *we have*

$$\begin{aligned} d\bigl((1-z)e\oplus z f, h\bigr) \leq (1-z)d(e, h) +z d (f, h). \end{aligned}$$

### Lemma 2.3

([21])

*Let* *X* *be a* $\operatorname{CAT}(0)$ *space*. *Then*

$$\begin{aligned} d\bigl((1 - z)e \oplus z f, h\bigr)^{2} \leq (1 - z)d(e, h)^{2} + zd(f, h)^{2}-z (1 - z)d(e, f)^{2} \end{aligned}$$

*for all* $e, f, h \in X$ *and* $z \in [0, 1]$.

Let $\{u_{n}\}$ be a bounded sequence in a complete $\operatorname{CAT}(0)$ space *X*. For $u \in X$, we denote

$$\begin{aligned} r\bigl(u, \{u_{n}\}\bigr)=\limsup_{n \to \infty } d(u, u_{n}). \end{aligned}$$

The asymptotic radius $r(\{u_{n}\})$ is given by

$$\begin{aligned} r\bigl(\{u_{n}\}\bigr)=\inf \bigl\{ r(u, u_{n}): u \in X\bigr\} , \end{aligned}$$

and the asymptotic center $A(\{u_{n}\})$ of $\{u_{n}\}$ is defined as

$$\begin{aligned} A\bigl(\{u_{n}\}\bigr)=\bigl\{ u \in X: r(u, u_{n})=r\bigl( \{u_{n}\}\bigr)\bigr\} . \end{aligned}$$

It is known that in a $\operatorname{CAT}(0)$ space, $A(\{u_{n}\})$ consists of exactly one point [25, Proposition 5].

In 1976, Lim [26] introduced the concept of Δ-convergence in a metric space. Later on, Kirk and Panyanak [22] proved that $\operatorname{CAT}(0)$ spaces presented a natural framework for Lim’s concept and provided precise analogs of several results in Banach spaces involving weak convergence in $\operatorname{CAT}(0)$ space setting.

### Definition 2.4

A sequence $\{u_{n}\}$ in *X* is said to be Δ-convergent to $u\in X$ if *u* is the unique asymptotic center of $\{v_{n}\}$ for every subsequence $\{v_{n}\}$ of $\{u_{n}\}$. In this case, we write $\Delta \text{-}\lim_{n} {u_{n}}=u$ and say that *u* is the Δ-limit of $\{u_{n}\}$.

### Definition 2.5

A Banach space *X* is said to satisfy Opial’s condition if for any sequence $\{u_{n}\}$ in *X* with $u_{n} \rightharpoonup u$ (⇀ denotes weak convergence), we have $\limsup_{n\to \infty } \|u_{n} - u\| < \limsup_{n\to \infty } \|u_{n} - v\|$ for all $v\in X$ with $v \neq u$.

Examples of Banach spaces satisfying this condition are Hilbert spaces and all $l^{p}$ spaces ($1 < p < \infty$). On the other hand, $L^{p} [0, 2\pi ]$ with $1 < p \neq 2$ fail to satisfy Opial’s condition.

Notice that if given a sequence $\{u_{n}\}$ in *X* such that $\{u_{n}\}$ Δ-converge to *u*, then for $v\in X$ with $v \neq u$, we have

$$\begin{aligned} \limsup_{n\to \infty } \Vert u_{n} - u\Vert < \limsup _{n\to \infty } \Vert u_{n} - v\Vert . \end{aligned}$$

So, every $\operatorname{CAT}(0)$ space satisfies Opial’s property.

### Lemma 2.6

([22])

*Every bounded sequence in a complete* $\operatorname{CAT}(0)$ *space admits a* Δ-*convergent subsequence*.

### Lemma 2.7

([21])

*If* *G* *is a closed convex subset of a complete* $\operatorname{CAT}(0)$ *space* *X* *and if* $\{u_{n}\}$ *is a bounded sequence in* *G*, *then the asymptotic center of* $\{u_{n}\}$ *is in* *G*.

Next, we introduce the concept of partial order in the setting of $\operatorname{CAT}(0)$ spaces.

Let *X* be a complete $\operatorname{CAT}(0)$ space endowed with partial order “⪯”. An order interval is any of the subsets

$$ [a, \rightarrow) = \{ u \in X; a\preceq u\} \quad \text{or} \quad ( \leftarrow, a]= \{ u\in X: u\preceq a \} $$

for any $a\in X$. So, an order interval $[u, v]$ for all $u, v\in X$ is given by

$$ [u, v] = \{ w\in X : u\preceq w \preceq v\}. $$

Throughout we will assume that the order intervals are closed and convex subsets of an ordered $\operatorname{CAT}(0)$ space $(X, \preceq)$.

### Definition 2.8

Let *G* be a nonempty subset of an ordered metric space *X*. A mapping $P:G \rightarrow G $ is said to be:

(i) monotone if $Pu \preceq Pv$ for all $u, v\in G$ with $u \preceq v$,
(ii) monotone nonexpansive if P is monotone and
  $$\begin{aligned} d(Pu, Pv) \leq d(u, v) \end{aligned}$$
  for all $u, v\in G$ with $u\preceq v$.

Now we present the Mann iteration scheme in the setting of ordered $\operatorname{CAT}(0)$ spaces $(X, \preceq)$. Let *G* be a nonempty convex subset of a $\operatorname{CAT}(0)$ space *X*. Then the Mann iteration is as follows:

2.1 $$\begin{aligned}& \begin{aligned} &u_{1} \in G, \\ &u_{n+1}= (1-\kappa_{n}) u_{n} \oplus \kappa_{n} Pu_{n},\quad n\in \mathbb{N,} \end{aligned} \end{aligned}$$

where $\{\kappa_{n}\} \subset [0, 1]$. In this paper, we prove some Δ-convergence and strong convergence results in $\operatorname{CAT}(0)$ spaces.

## Some Δ-convergence and strong convergence theorems

We begin with the following important lemma.

### Lemma 3.1

*Let* *G* *be a nonempty closed convex subset of a complete ordered* $\operatorname{CAT}(0)$ *space* $(X,\preceq)$, *and let* $P : G \to G$ *be a monotone nonexpansive mapping*. *Fix* $u_{1}\in G$ *such that* $u_{1} \preceq Pu_{1}$. *If* $\{u_{n}\}$ *is defined by* (2.1) *with condition* $\sum_{n=1} ^{\infty }\kappa_{n} (1-\kappa_{n})=\infty $, *then we have*:

(i) $u_{n} \preceq u_{n+1} \preceq Pu_{n}$ *for any* $n\geq 1$,
(ii) $u_{n} \preceq u$, *provided that* $\{u_{n}\}$ Δ-*converges to a point* $u\in G $.

### Proof

(i) We will prove the result by induction on *n*. Note that if $q_{1}, q_{2} \in G $ are such that $q_{1}\preceq q_{2}$, then $q_{1} \preceq \lambda q_{1} + (1-\lambda)q_{2} \preceq q_{2}$ for any $\lambda \in [0, 1] $. This is true because we have assumed that order intervals are convex. Thus we only need to show that $u_{n} \preceq Pu_{n} $ for any $n\geq 1 $. We have already assumed that $u_{1} \preceq Pu_{1}$, and hence the inequality holds for $n=1$. Assume that $u_{n} \preceq Pu_{n} $ for $n\geq 2 $. Since $\kappa_{n} \in [0, 1] $ for all *n*, we have

$$\begin{aligned} u_{n} \preceq (1- \kappa_{n})u_{n} \oplus \kappa_{n}Pu_{n} \preceq Pu _{n}, \end{aligned}$$

that is, $u_{n} \preceq u_{n+1} \preceq Pu_{n} $. Since *P* is monotone, we have $Pu_{n} \preceq Pu_{n+1}$. By using the transitivity of the order we get $u_{n+1} \preceq Pu_{n+1} $. Thus by induction the inequality is true for any $n \geq 1 $.

(ii) Let *u* be the Δ-limit of $\{u_{n}\}$. From part (i) we have $u_{n} \preceq u_{n+1} $ for all $n \geq 1$ since $\{u_{n}\}$ is increasing and the order interval $[u_{m}, \rightarrow)$ is closed and convex. Therefore $u \in [u_{m}, \rightarrow) $ for a fixed $m \in \mathbb{N} $; otherwise, if $u \notin [u_{m}, \rightarrow) $, then we could construct a subsequence $\{u_{r}\}$ of $\{u_{n}\}$ by leaving the first $m-1$ terms of the sequence $\{u_{n}\}$, and then the asymptotic center of $\{u_{r}\}$ would not be *u*, which contradicts the assumption that *u* is the Δ-limit of the sequence $\{u_{n}\}$. This completes the proof of part (ii). □

### Lemma 3.2

*Let* *G* *be a nonempty closed convex subset of a complete* $\operatorname{CAT}(0)$ *space* $(X,\preceq)$, *and let* $P : G \to G$ *be a monotone nonexpansive mapping*. *Fix* $u_{1}\in G$ *such that* $u_{1} \preceq Pu_{1}$. *If* $\{u_{n}\}$ *is a sequence described as in* (2.1) *and* $F(P) \neq \emptyset $ *with* $r \in F(P)$ *such that* $r \preceq u_{1}$, *then*:

(i) $\lim_{n\to \infty } d(u_{n}, r)$ *exists*, *and*
(ii) $\lim_{n\to \infty } d(Pu_{n},u_{n})=0$.

### Proof

(i) Since $r \preceq u_{1}$, using part (i) of Lemma 3.1, we have $u_{n} \preceq u_{n+1} \preceq Pu_{n}$. In particular, for $n=1$, we have $u_{1} \preceq u_{2} \preceq Pu_{1}$. Using the transitivity of the order, we get $r \preceq u_{2}$. By mathematical induction we have $r \preceq u_{n}$ for all $n\geq 1$. Now we have

$$\begin{aligned} d(u_{n+1}, r) =& d\bigl((1-\kappa_{n})u_{n} \oplus \kappa_{n}Pu_{n},r\bigr) \\ \leq & (1-\kappa_{n})d(u_{n},r)+\kappa_{n}d(Pu_{n},r) \\ =& (1-\kappa_{n})d(u_{n},r)+\kappa_{n}d(Pu_{n}, Pr). \end{aligned}$$

Since *P* is a monotone map and $r \preceq u_{n}$ for all $n \geq 1$, we have

$$\begin{aligned} d(u_{n+1}, r) \leq & (1-\kappa_{n})d(u_{n}, r)+ \kappa_{n}d(u_{n}, r) \\ =& d(u_{n}, r). \end{aligned}$$

Thus we have $d(u_{n+1}, r) \leq d(u_{n}, r) $ for all $n \geq 1$. So $\{d(u_{n}, r)\}$ is a decreasing real sequence bounded below by zero. Hence $\lim_{n\to \infty } d(u_{n}, r)$ exists.

(ii) First, consider

$$\begin{aligned} d(Pu_{n+1}, u_{n+1}) =& d\bigl(Pu_{n+1}, (1- \kappa_{n})u_{n} \oplus \kappa _{n}Pu_{n} \bigr) \\ \leq & (1-\kappa_{n})d(Pu_{n+1}, u_{n}) + \kappa_{n}d(Pu_{n+1}, Pu _{n}) \\ \leq & (1-\kappa_{n})d(Pu_{n+1}, u_{n}) + \kappa_{n}d(u_{n+1}, u_{n}) \\ \leq & (1-\kappa_{n}) \bigl(d(Pu_{n+1}, Pu_{n}) + d(Pu_{n}, u_{n})\bigr) + \kappa_{n}d(u_{n+1}, u_{n}) \\ \leq & (1-\kappa_{n}) \bigl(d(u_{n+1}, u_{n}) + d(Pu_{n}, u_{n})\bigr) + \kappa _{n}d(u_{n+1}, u_{n}) \\ =& (1-\kappa_{n})d(Pu_{n}, u_{n}) + d(u_{n+1}, u_{n}) \\ =& (1-\kappa_{n})d(Pu_{n}, u_{n}) + d\bigl((1- \kappa_{n})u_{n} \oplus \kappa_{n}Pu_{n}, u_{n}\bigr) \\ \leq & (1-\kappa_{n})d(Pu_{n}, u_{n}) + (1- \kappa_{n})d(u_{n},u_{n})+ \kappa_{n}d(Pu_{n}, u_{n}) \\ =& d(Pu_{n}, u_{n}). \end{aligned}$$

So $\lim_{n\to \infty } d(Pu_{n},u_{n})$ exists.

Since $r\preceq u_{1}$, using the Lemma 3.1, we have $r\preceq u_{1} \preceq u_{n}$ for all $n\geq 1$. Then, since *P* is a nonexpansive map and *r* is a fixed point of *P*, we have

$$\begin{aligned} d(u_{n+1}, r)^{2} =& d\bigl((1-\kappa_{n})u_{n} \oplus \kappa_{n} Pu_{n}, r\bigr)^{2} \\ \leq & (1-\kappa_{n}) d(u_{n},r)^{2}+ \kappa_{n} d(Pu_{n},r)^{2}-(1- \kappa_{n}) \kappa_{n} d(u_{n}, Pu_{n})^{2} \\ =& (1-\kappa_{n}) d(u_{n},r)^{2}+ \kappa_{n} d(Pu_{n},Pr)^{2}-(1- \kappa_{n})\kappa_{n} d(u_{n}, Pu_{n})^{2} \\ \leq & (1-\kappa_{n}) d(u_{n},r)^{2}+ \kappa_{n} d(u_{n},r)^{2}-(1- \kappa_{n}) \kappa_{n} d(u_{n}, Pu_{n})^{2} \\ =& d(u_{n},r)^{2}-(1-\kappa_{n}) \kappa_{n} d(u_{n}, Pu_{n})^{2}. \end{aligned}$$

From this we get

3.1 $$\begin{aligned} \sum_{n=1}^{\infty }(1-\kappa_{n}) \kappa_{n} d(u_{n}, Pu_{n})^{2} \leq d(u_{1},r)^{2} < \infty. \end{aligned}$$

Since $\sum_{n=1}^{\infty }(1-\kappa_{n})\kappa_{n} = \infty $, there exists a subsequence $\{u_{n_{k}}\}$ of $\{u_{n}\}$ such that

$$\begin{aligned} \lim_{n\to \infty } d(Pu_{n_{k}},u_{n_{k}})=0. \end{aligned}$$

Since $\lim_{n\to \infty } d(Pu_{n},u_{n})$ exists, it follows that $\lim_{n\to \infty } d(Pu_{n},u_{n})=0$, and this proves the result. □

The following lemma is an analogue of Theorem 3.7 of [22].

### Lemma 3.3

*Let* *G* *be a nonempty closed convex subset of a complete* $\operatorname{CAT}(0)$ *space* $(X,\preceq)$, *and let* $P : G \to G$ *be a monotone nonexpansive mapping*. *Fix* $u_{1}\in G$ *such that* $u_{1} \preceq Pu_{1}$. *If* $\{u_{n}\}$ *is a sequence described as in* (2.1), *then the conditions* $\Delta \text{-}\lim_{n} {u_{n}}=u$ *and* $\lim_{n\to \infty } d(Pu_{n}, u_{n})=0$ *imply that* *u* *is a fixed point of* *P*.

### Proof

Since Δ-$\lim_{n} {u_{n}}=u$, by Lemma 3.1 we get $u_{n} \preceq u$ for all $n\geq 1$. Then from the nonexpansiveness of *P* and $\lim_{n\to \infty } d(Pu_{n}, u_{n})=0$ it follows that

$$\begin{aligned}& d(Pu, u_{n}) \leq d(Pu, Pu_{n})+d(Pu_{n}, u_{n}), \\& \limsup_{n\to \infty } d(Pu, u_{n}) \leq \limsup _{n\to \infty }\bigl[ d(Pu, Pu_{n})+d(Pu_{n}, u_{n})\bigr] \\& \hphantom{\limsup_{n\to \infty } d(Pu, u_{n})}= \limsup_{n\to \infty } d(Pu, Pu_{n}) \\& \hphantom{\limsup_{n\to \infty } d(Pu, u_{n})}\leq \limsup_{n\to \infty } d(u, u_{n}). \end{aligned}$$

Thus by the uniqueness of asymptotic center we get $Pu=u$, which proves the desired result. □

### Theorem 3.4

*Let* *G* *be a nonempty closed convex subset of a complete* $\operatorname{CAT}(0)$ *space* $(X,\preceq)$, *and let* $P : G \to G$ *be a monotone nonexpansive mapping with* $F(P)\neq \emptyset $. *Fix* $u_{1}\in G$ *such that* $u_{1} \preceq Pu_{1}$. *If* $\{u_{n}\}$ *is a sequence described as in* (2.1), *then* $\{u_{n}\}$ Δ-*converges to a fixed point of* *P*.

### Proof

From Lemma 3.2 we have that $\lim_{n\to \infty } d(u_{n}, r)$ exists for each $r\in F(P)$, so the sequence $\{u_{n}\}$ is bounded, and $\lim_{n\to \infty }d(u_{n}, Pu_{n})=0 $.

Let $W_{\omega }(\{u_{n}\})=: \bigcup X(\{v_{n}\})$, where the union is taken over all subsequences $\{v_{n}\}$ over $\{u_{n}\}$. To show the Δ-convergence of $\{u_{n}\}$ to a fixed point of *P*, we will first prove that $W_{\omega }(\{u_{n}\}) \subset F(P)$ and thereafter argue that $W_{\omega }(\{u_{n}\})$ is a singleton set. To show that $W_{\omega }(\{u_{n}\}) \subset F(P)$, let $y\in W_{\omega }(\{u_{n} \})$. Then there exists a subsequence $\{y_{n}\}$ of $\{u_{n}\}$ such that $X(\{y_{n}\})=y$. By Lemmas 2.6 and 2.7 there exists a subsequence $\{z_{n}\}$ of $\{y_{n}\}$ such that $\Delta \text{-}\lim_{n} z_{n}=z$ and $z\in G$. Since $\lim_{n\to \infty } d(Pu _{n}, u_{n})=0$ and $\{z_{n}\}$ is a subsequence of $\{u_{n}\}$, we have that $\lim_{n\to \infty } d(z_{n}, Pz_{n})=0$. In view of Lemma 3.3, we have $z=Pz$, and hence $z\in F(P)$.

Now we wish to show that $z=y$. If, on the contrary, $z \neq y $, then we would have

$$\begin{aligned} \limsup_{n\to \infty } d(z_{n}, z) < &\limsup _{n\to \infty } d (z_{n}, y) \\ \leq &\limsup_{n\to \infty } d(y_{n}, y) \\ < & \limsup_{n\to \infty } d(y_{n}, z) \\ =& \limsup_{n\to \infty } d(u_{n}, z) \\ =& \limsup_{n\to \infty } d(z_{n}, z), \end{aligned}$$

which is a contradiction since *X* satisfies the Opial condition and hence $z=y\in F(P)$. Now it remains to show that $W_{\omega }(\{u_{n} \})$ consists of a single element only. For this, let $\{y_{n}\}$ be a subsequence of $\{u_{n}\}$. Again, using Lemmas 2.6 and 2.7, we can find a subsequence $\{z_{n}\}$ of $\{y_{n}\}$ such that Δ-$\lim_{n} z_{n}=z$. Let $X(\{y_{n}\})=y$ and $X(\{u_{n}\})=u$. Previously, we have already proved that $y=z$; therefore, it suffices to show that $z=u$. If $z \neq u$, then since $z\in F(P)$, $\{d(u_{n}, z)\}$ is convergent by Lemma 3.2, By the uniqueness of asymptotic center we have

$$\begin{aligned} \limsup_{n\to \infty } d(z_{n}, z) < & \limsup _{n\to \infty } d(z_{n}, u) \\ \leq & \limsup_{n\to \infty } d(u_{n}, u) \\ < & \limsup_{n\to \infty } d(u_{n}, z) \\ =&\limsup_{n\to \infty } d(z_{n}, z), \end{aligned}$$

which gives a contradiction. Therefore we must have $z=u$, which proves that $W_{\omega }(\{u_{n}\})$ is a singleton set and that a particular element is a fixed point of *P*. Hence the conclusion follows. □

### Theorem 3.5

*Let* *X* *be a complete* $\operatorname{CAT}(0)$ *space endowed with partial ordering* ′⪯′, *and let* *G* *be a nonempty closed convex subset of* *X*. *Let* $P : G \to G$ *be a monotone nonexpansive mapping such that* $F(P) \neq \emptyset $. *Fix* $u_{1}\in G$ *such that and* $u_{1} \preceq Pu _{1}$. *If* $\{u_{n}\}$ *is a sequence described as in* (2.1) *such that* $\sum_{n=1}^{\infty }\kappa_{n} (1-\kappa_{n})=\infty $, *then* $\{u_{n}\}$ *converges to a fixed point of* *P* *if and only if* $\liminf_{n\to \infty } d(u_{n}, F(P))=0$.

### Proof

If the sequence $\{u_{n}\}$ converges to a point $u\in F(P)$, then it is obvious that $\liminf_{n\to \infty } d(u_{n},F(P))=0$.

For the converse part, assume that $\liminf_{n\to \infty } d(u _{n}, F(P))=0$. From Lemma 3.2(i) we have

$$\begin{aligned} d(u_{n+1},r) \leq d(u_{n},r) \quad \text{for any } r\in F(P), \end{aligned}$$

so that

$$\begin{aligned} d\bigl(u_{n+1},F(P)\bigr) \leq d\bigl(u_{n},F(P)\bigr). \end{aligned}$$

Thus $\{d(u_{n},F(P))\}$ forms a decreasing sequence that is bounded below by zero, so $\lim_{n\to \infty } d(u_{n}, F(P))$ exists. As $\liminf_{n\to \infty } d(u_{n}, F(P))=0$, we have $\lim_{n\to \infty } d(u_{n}, F(P))=0$.

Now we prove that $\{u_{n}\}$ is a Cauchy sequence in *G*. Let *ϵ*>0 be arbitrary. Since $\liminf_{n\to \infty } d(u _{n}, F(P))=0$, there exists $n_{0}$ such that, for all $n \geq n_{0}$, we have

$$\begin{aligned} d\bigl(u_{n}, F(P)\bigr) < \frac{\epsilon }{4}. \end{aligned}$$

In particular,

$$\begin{aligned} \inf \bigl\{ d(u_{n_{0}}, r): r\in F(P) \bigr\} < \frac{\epsilon }{4}, \end{aligned}$$

so there must exist $r \in F(P)$ such that

$$\begin{aligned} d(u_{n_{0}},r) < \frac{\epsilon }{2}. \end{aligned}$$

Thus, for $m, n \geq n_{0}$, we have

$$\begin{aligned} d(u_{n+m}, u_{n}) \leq d(u_{n+m}, r) + d( u_{n}, r)< 2 d( u_{n_{0}}, r) < 2\frac{\epsilon }{2} = {\epsilon,} \end{aligned}$$

which shows that $\{u_{n}\}$ is a Cauchy sequence. Since *G* is a closed subset of a complete metric space *X*, so *G* itself is a complete metric space, and therefore $\{u_{n}\}$ must converge in *G*. Let $\liminf_{n\to \infty } u_{n} = q$.

Now *P* is a monotone nonexpansive mapping, and from Lemma 3.3(i) we have $\lim_{n\to \infty } d(Pu_{n},u_{n})=0$. Also, from the proof of Lemma 3.1 in [12] we can easily deduce that $u_{n} \preceq q$ for any $n \geq 1$. Therefore we have

$$\begin{aligned} d(q, Pq) \leq & d(q,u_{n}) + d(u_{n}, Pu_{n}) + d(Pu_{n}, Pq) \\ \leq & d(q, u_{n}) +d\bigl(u_{n}, P(u_{n})\bigr) + d(u_{n}, q) \\ \rightarrow & 0 \quad \text{as } n \rightarrow \infty, \end{aligned}$$

and hence $q = Pq$. Thus $q \in F(P)$. □

## Numerical example

In this section, we present a numerical example to illustrate the convergence behavior of our iteration scheme (2.1).

Let $X = [0, +\infty)$ be a complete metric space with the metric

$$\begin{aligned} d(u, v) = \vert u - v\vert ,\quad u, v \in X. \end{aligned}$$

Now, consider the order relation $u \preceq v$ as

$$\begin{aligned}& u, v \in [0, 1] \quad \text{and}\quad u \leq v\quad \text{or} \\& u, v \in (n, n+1] \quad \text{for some } n=1,2, \dots \quad \text{and}\quad u \leq v. \end{aligned}$$

Let *P* be defined by

$$\begin{aligned} P(0) = 0,\qquad P(u)=\frac{n}{2}+\frac{u}{2},\quad u \in (n, n+1], n = 0, 1, 2, \dots. \end{aligned}$$

Then, clearly, *P* is not continuous at $v= n+1$ for $n = 0, 1, 2, \dots$, since

$$\begin{aligned} P\bigl(n+1^{-}\bigr) = n+ \frac{1}{2} \neq n+1 = P \bigl(n+1^{+}\bigr). \end{aligned}$$

Also, if $u \succeq v$, then $u, v \in [0, 1]$ or $u, v \in (n, n+1]$ for some $n = 1, 2, \dots $, and

$$\begin{aligned} d\bigl(P(u), P(v)\bigr) = d\biggl(\frac{n}{2}+\frac{u}{2}, \frac{n}{2}+\frac{v}{2}\biggr)= \frac{1}{2}d(u, v). \end{aligned}$$

So, *P* is a monotone nonexpansive map but not a nonexpansive map, and 0 is the unique fixed point of *P*.

Now, we show the convergence of (2.1) using two different sets of values.

**Figure 1:**
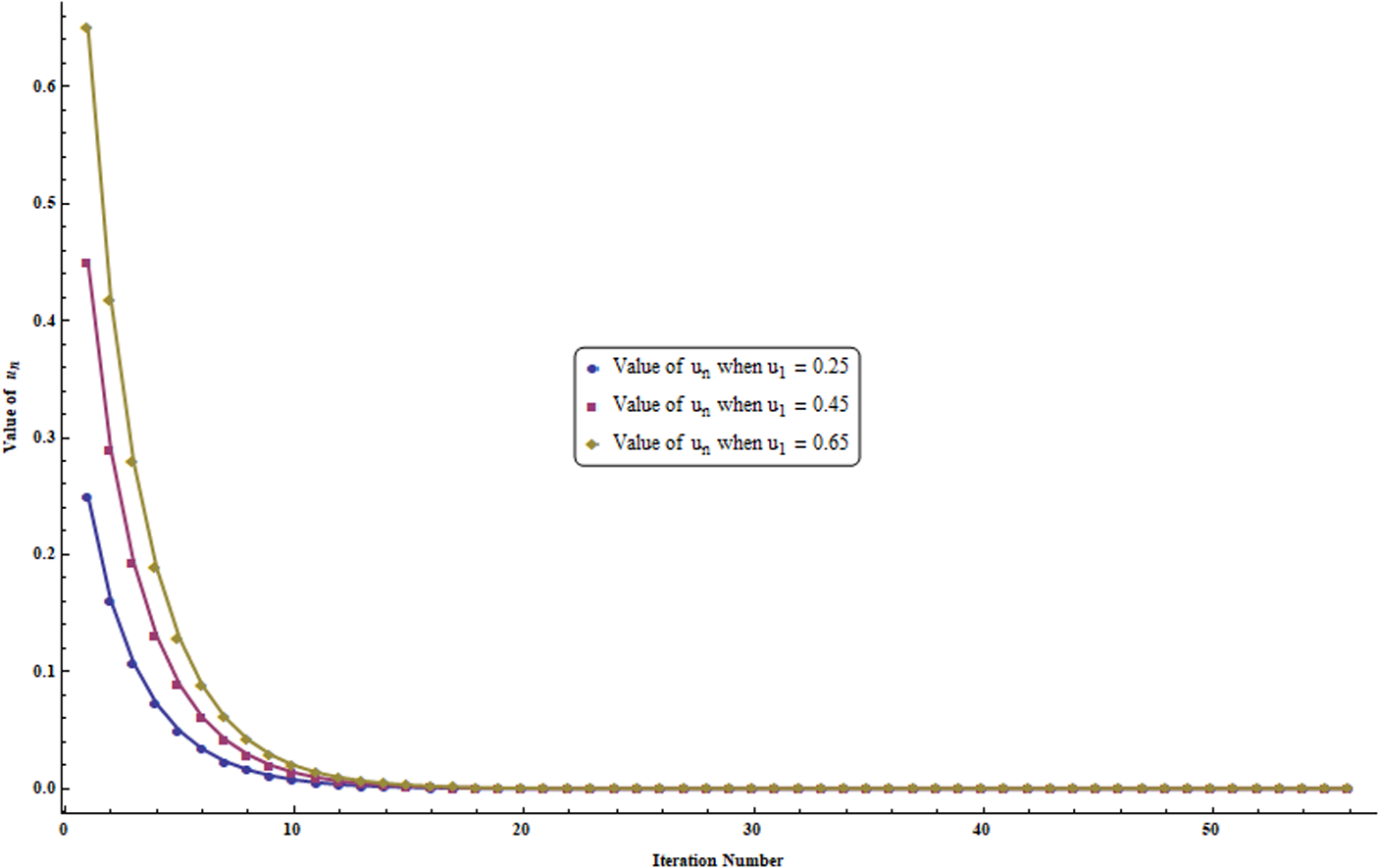
Graph corresponding to Table 1

**Figure 2:**
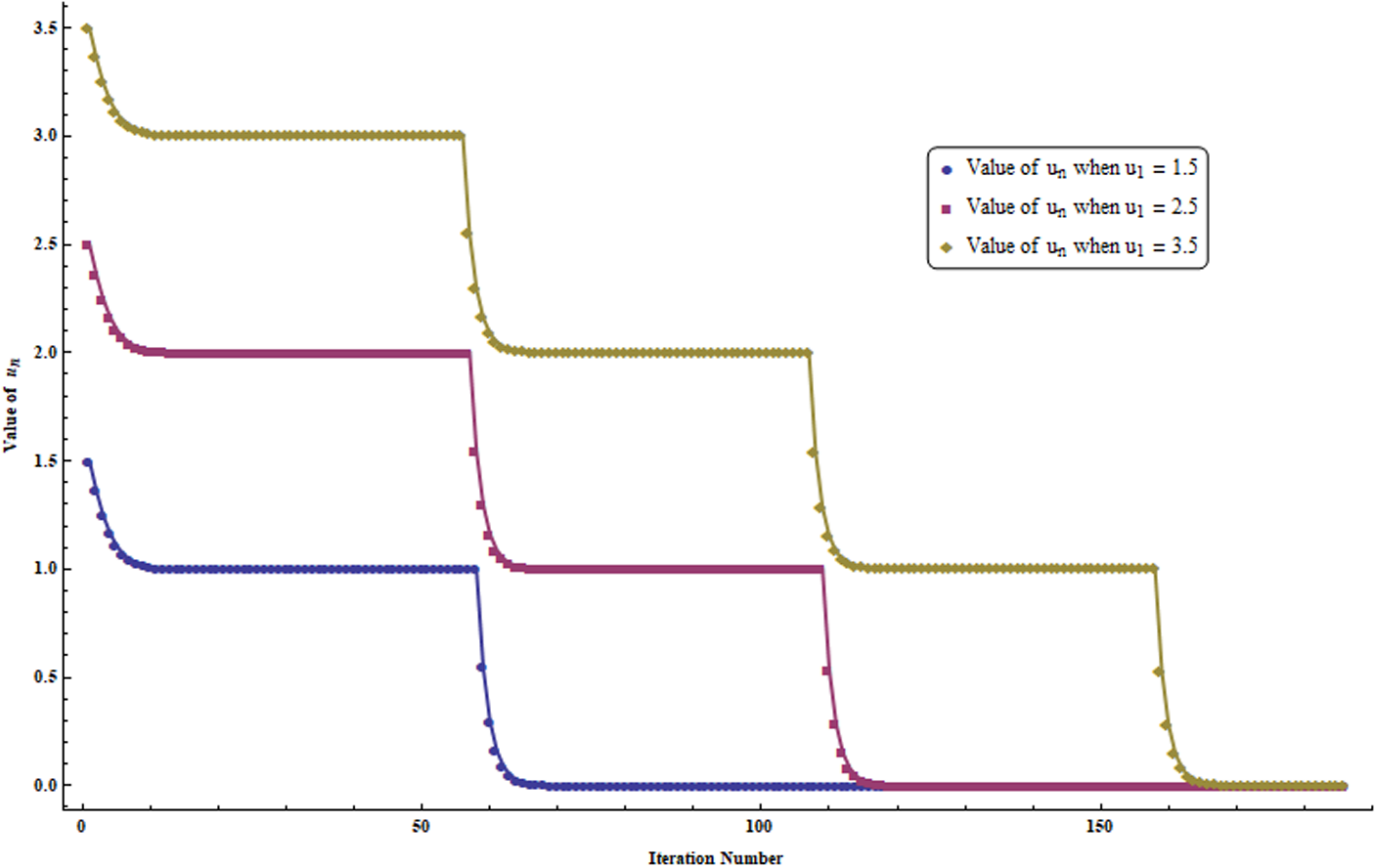
Graph corresponding to Table 2

It is evident from the tables (Table 1 and Table 2) and graphs (Fig. 1 and Fig. 2) that our sequence (2.1) converges to 0, which is a fixed point of *P*.

**Table 1:** ($\kappa_{n} = \frac{2n}{5n+2}$ for all $n \in \mathbb{N}$)

Step	When $u_{1} = 0.25$	$u_{1} = 0.45$	$u_{1} = 0.65$
1	0.25	0.45	0.65
2	0.1607142857142857	0.2892857142857142	0.4178571428571429
3	0.1071428571428571	0.1928571428571428	0.2785714285714286
4	0.07247899159663865	0.1304621848739496	0.1884453781512605
5	0.04941749427043545	0.0889514896867838	0.1284854851031322
6	0.03386013496307614	0.06094824293353705	0.088036350903998
7	0.02327884278711485	0.04190191701680672	0.0605249912464986
8	0.01604352678571429	0.02887834821428571	0.04171316964285715
9	0.01107767325680272	0.0199398118622449	0.02880195046768708
10	0.007660093209491246	0.01378816777708424	0.01991624234467724
11	0.005303141452724708	0.00954565461490447	0.01378816777708424
12	0.003674983989168876	0.006614971180503976	0.00955495837183908
13	0.002548779218294543	0.004587802592930178	0.006626825967565813
14	0.001768928860458153	0.003184071948824675	0.004599215037191199
15	0.001228422819762606	0.002211161075572691	0.003193899331382777
16	0.000853514556588304	0.001536326201858948	0.002219137847129592
17	0.0005932967039699188	0.001067934067145854	0.00154257143032179
18	0.0004125798918411505	0.0007426438053140709	0.001072707718786992
19	0.0002870120986721047	0.0005166217776097884	0.0007462314565474726
20	0.0001997249140244027	0.0003595048452439249	0.0005192847764634474
21	0.0001390242048601234	0.0002502435687482223	0.0003614629326363212
22	0.0000967972267484037	0.0001742350081471267	0.0002516727895458498
23	0.00006741235434263828	0.0001213422378167489	0.0001752721212908597
24	0.0000469581784523506	0.0000845247212142311	0.0001220912639761116
25	0.00003271676367581804	0.0000588901746164725	0.000085063585557127
26	0.00002279868964810942	0.00004103764136659699	0.00005927659308508453
27	0.000015889995815349	0.00002860199246762819	0.00004131398911990741
28	0.00001107660292237831	0.00001993788526028096	0.00002879916759818363
29	7.722420347291922 × 10^−6^	0.00001390035662512546	0.00002007829290295901
30	5.384680854404231 × 10^−6^	9.69242553792 × 10^−6^	0.00001400017022145
31	3.755106385308214 × 10^−6^	6.759191493554787 × 10^−6^	9.76327660180 × 10^−6^

**Table 2:** ($\kappa_{n} = {\sqrt{\frac{n}{2n+3}}}$ for all $n \in \mathbb{N} $)

Step	When $u_{1} = 1.5$	$u_{1} = 2.5$	$u_{1} = 3.5$
1	1.5	2.5	3.5
50	1.000070736246516	2.000070736246516	3.000070736246516
100	1.000000020810691	2.000000020810692	3.000000020810691
150	1.000000000006688	2.000000000006688	3.000000000006689
200	0.1721565830342946	1.090775274459172	2.056164187502003
250	0.00005853496173392133	1.00003086450209	2.000019096386024
300	2.015550211966978 × 10^−8^	1.000000010627658	2.000000006575511
350	7.001008916808291 × 10^−12^	1.000000000003692	2.000000000002284
400	2.447383506364153 × 10^−15^	0.0918987936870247	1.030161439675001
450	8.59732107577053 × 10^−19^	0.00003228278010981194	1.000010595298216
500	3.031773634861876 × 10^−22^	1.13842533894431 × 10^−8^	1.000000003736344
550	1.072466692421632 × 10^−25^	4.027092404879379 × 10^−12^	1.000000000001322
600	3.803545320138658 × 10^−29^	1.42822416570892 × 10^−15^	0.03556653915576083
650	1.351861194143804 × 10^−32^	5.076213541974867 × 10^−19^	0.00001264110719020337

## Application to integral equations

In this section, we use our iteration scheme (2.1) to find the solution of following integral equation:

IE $$ u(t) = h(t) + \int_{0}^{1}B\bigl(t, v, u(v)\bigr)\,dv,\quad t \in [0, 1], $$

where

(i) $h\in L^{2} ([0, 1], \mathbb{R})$,
(ii) $B: [0,1] \times [0, 1] \times L^{2} ([0, 1], \mathbb{R}) \rightarrow \mathbb{R}$ is measurable and satisfies the condition
  $$\begin{aligned} 0 \leq \bigl\vert B(t, v, u) - B(t, v, w)\bigr\vert \leq \Vert u - w \Vert \end{aligned}$$
  for $t, v \in [0, 1]$ and $u, w \in L^{2} ([0, 1], \mathbb{R})$ such that $u \leq w$.

Recall that, for all $u, w \in L^{2}([0, 1], \mathbb{R})$, we have

$$\begin{aligned} u \leq w\quad \Leftrightarrow\quad u(t) \leq w(t) \quad \text{for almost every } t \in [0, 1]. \end{aligned}$$

Next, assume that there exist a nonnegative function $f(\cdot,\cdot) \in L^{2}([0, 1] \times [0, 1])$ and $M < \frac{1}{2}$ such that

$$\begin{aligned} \bigl\vert B(t, v, u)\bigr\vert \leq f(t, v) + M\vert u\vert \end{aligned}$$

for $t, v \in [0, 1]$ and $u \in L^{2}([0, 1], \mathbb{R})$.

Let

$$\begin{aligned} G = \bigl\{ w \in L^{2}\bigl([0, 1], \mathbb{R}\bigr) \text{ such that } \Vert w\Vert _{L^{2}([0, 1], \mathbb{R})} \leq \rho \bigr\} , \end{aligned}$$

where *ρ* is sufficiently large, that is, *G* is the closed ball of $L^{2}([0, 1], \mathbb{R})$ centered at 0 with radius *ρ*.

Define the operator $P: L^{2}([0, 1], \mathbb{R}) \rightarrow L^{2}([0, 1], \mathbb{R})$ by

5.1 $$\begin{aligned} P\bigl(w(t)\bigr) = h(t) + \int_{0}^{1}B\bigl(t, v, w(v)\bigr)\,dv. \end{aligned}$$

Then $P(G)\subset G$, and it is a monotone nonexpansive map.

It is worth mentioning that every Hilbert space is a $\operatorname{CAT}(0)$ space, and so is $L^{2}([0, 1], \mathbb{R})$. Taking $X = L^{2}([0, 1], \mathbb{R})$ and *P* as in (5.1) in Theorem 3.4, we get the following result.

### Theorem 5.1

*Under the above assumptions*, *the sequence generated by iteration scheme* (2.1) *converges to a solution of integral equation* (IE).
